# Supplementary figures and images for: Metastability and teleconnection of atmospheric circulation via hidden Markov models and network modularity
Source: Sci Rep. 2025 Sep 30;15:34095. doi: 10.1038/s41598-025-14696-4 (PMC12484855; doi:10.1038/s41598-025-14696-4)

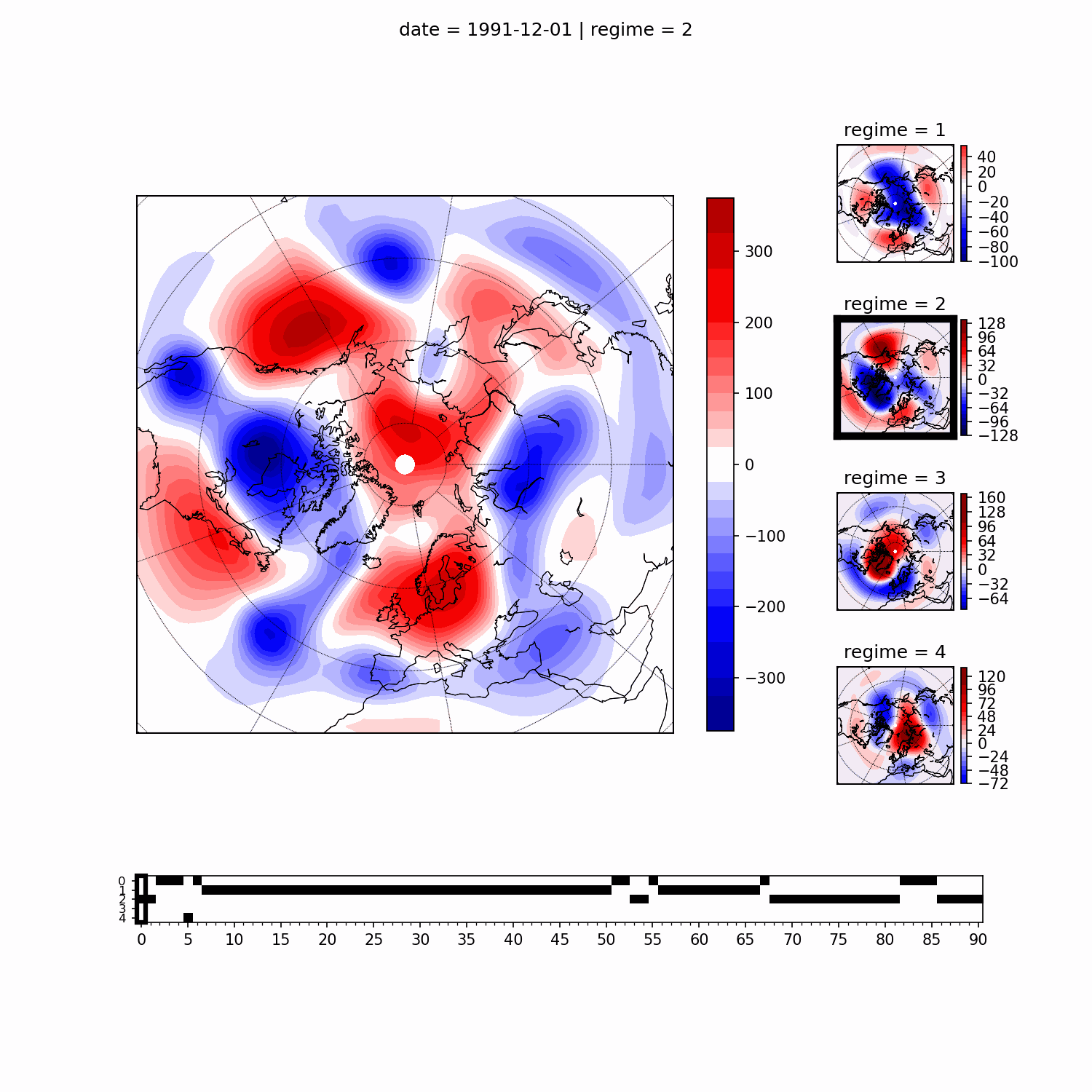

Supplement: Supplementary file 1 — Supplementary Information 1. [file 41598_2025_14696_MOESM1_ESM.gif]

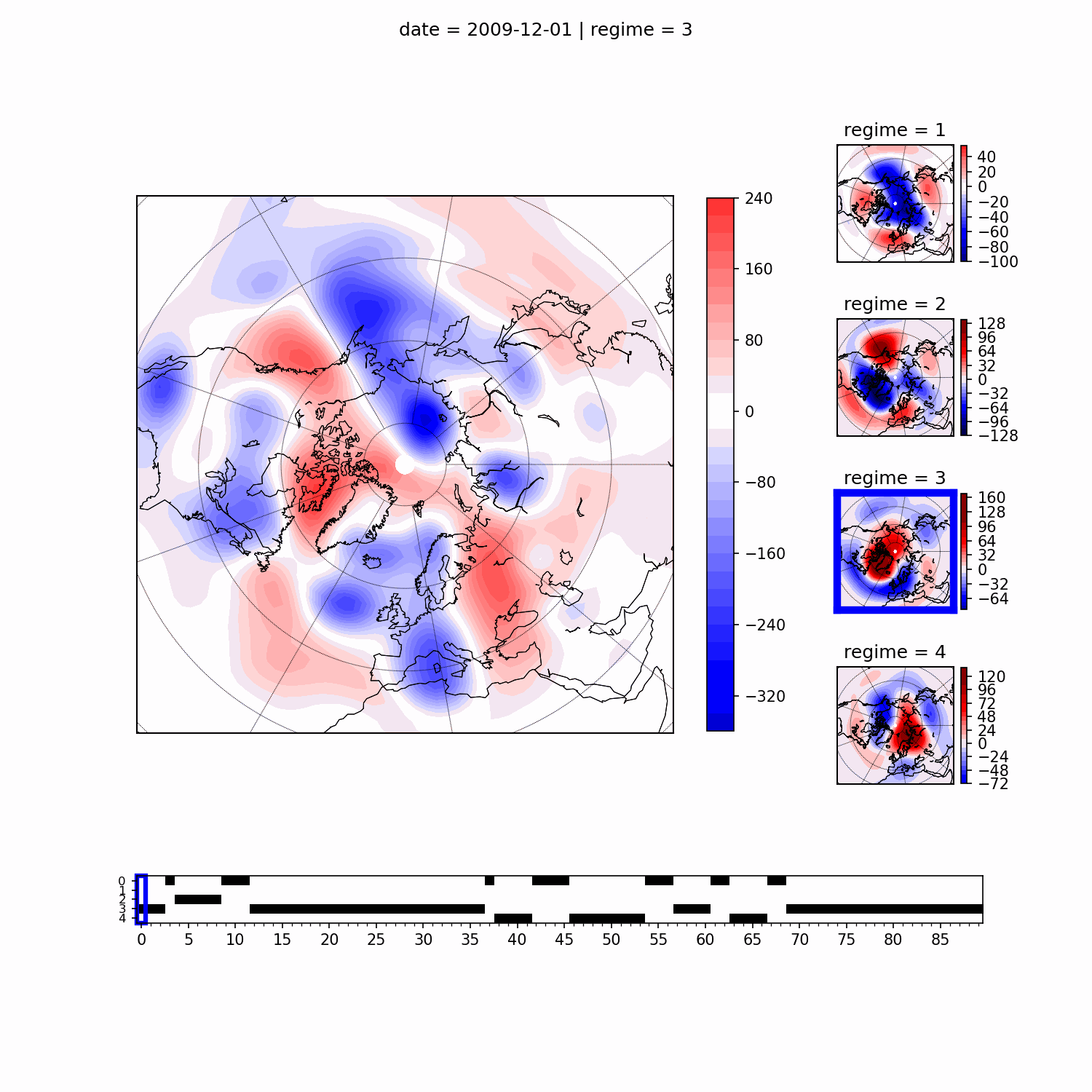

Supplement: Supplementary file 2 — Supplementary Information 2. [file 41598_2025_14696_MOESM2_ESM.gif]
