# Supplementary material for: Metastability and teleconnection of atmospheric circulation via hidden Markov models and network modularity
Source: Sci Rep. 2025 Sep 30;15:34095. doi: 10.1038/s41598-025-14696-4 (PMC12484855; doi:10.1038/s41598-025-14696-4)
Supplement: Supplementary file 3 — Supplementary Information 3. [file 41598_2025_14696_MOESM3_ESM.pdf]

Supplementary Information

Metastability and teleconnection of atmospheric circulation via hidden Markov models and network modularity

Dmitry Mukhin[1,*], **Roman Samoilov**[1], **and Abdel Hannachi**[2]

[1]Institute of Applied Physics of RAS, Nizhny Novgorod, 603950, Russia
[2]Stockholm University, Department of Meteorology, Stockholm, SE-106 91, Sweden
[*]mukhin@ipfran.ru

Supplementary text

Significance testing According to the method described in Sec. 2.2 of the main article, a split of a community A of HMM hidden states can be realized only if it provides positive increment ΔM_A of the modularity function M. However, to ensure this increment is significant, we need to reject the null hypothesis that the split yields the same increment by chance, i.e. for a sample taken from a random process without metastable regimes. In order to make the test stronger, we require the random process to have the same invariant distribution as that of the original HMM process and with similar spectral properties on short timescales. We use an ensemble approach to formulate the null hypothesis by using samples generated by means of the original HMM operator, but with shuffled time blocks. Using the original HMM as a generator of surrogates guarantees preserving the invariant measure, whereas block shuffling destroys autocorrelations crucial for persistent regimes.

The important hyperparameter of this procedure is block length, which determines autocorrelation properties of surrogates. For example, primitive shuffling with unit blocks corresponds to testing against a white noise process. In order to make the spectrum of the surrogates closer to the spectrum of the original HMM process at short time scales, we use distribution of block lengths derived from the HMM transition matrix decomposition. The evolution of an initial distribution $\mathbf{q}$ over discrete time t under the iterated Markov operator $\mathbf{Q}^t$ can be decomposed using the eigenbasis of the stochastic matrix $\mathbf{Q}$ columns as

$$\mathbf{Q}^t\mathbf{q} = \pi + a_2\lambda_2^t\mathbf{u}_2 + a_3\lambda_3^t\mathbf{u}_3 + \ldots, \tag{S1}$$

where $1 > \lambda_2 > \lambda_3 > \ldots$ are eigenvalues of the stochastic matrix $\mathbf{Q}$, with respective right eigenvectors $\pi, \mathbf{u}_2, \mathbf{u}_3, \ldots$ where π is the stationary distribution, and a_i is a coordinate of $\mathbf{q}$ along the eigenvector $\mathbf{u}_i$. This decomposition shows that during convergence of an initial distribution $\mathbf{q}$ to the stationary distribution π, different components of $\mathbf{q}$ vanish with different e-folding times $T_i = 1/\log|\lambda_i|$. This spectrum $\Theta = \{T_2, T_3, \ldots\}$ of characteristic times is then used as a distribution for the block lengths to generate surrogates.

Technically, a model time series is generated by iterating the Markov chain of hidden state probabilities inside the HMM and sampling the emission PDFs at each iteration to obtain a state in the KPC space. To generate a shuffled time series, we randomly draw T_i from Θ, iterate the HMM T_i times, and then randomize the Markov chain using new initial conditions from the stationary distribution π. A new value from Θ is then drawn and the HMM iterated again, etc. The procedure continues until the required time series length is reached.

For the significance test we use an ensemble of 10,000 surrogate times series, and from each of these we obtain a surrogate transition probability matrix, given the fixed emission PDF parameters taken from the original HMM. When splitting a current community A (see Sec. 2.2) into two new communities in the original HMM, we also measure the modularity increment ΔM from this split in each surrogate. The obtained ensemble of increments provides us with a null hypothesis distribution of ΔM, which is used to decide on the acceptance/rejection of the split. A ΔM below a significance level 1% is treated as non significant and the corresponding split rejected.

Regimes of QG3 model Quasi-geostrophic (QG) models of the atmosphere are widely used for simulating realistic mid-latitude atmosphere behavior, see e.g.[1–4] QG models, which are based on quasi-geostrophic potential vorticity conservation, demonstrate a rich spectrum of variability at different time scales and are competitive to intermediate and full general circulation models regarding complexity and dynamical features/processes. Here we use several time series generated by a three-level QG model (QG3) on the sphere[5] with realistic orography and surface boundary condition. Based on the equations of the potential vorticity at three (200, 500, and 800 hPa) pressure levels,[5–7] the model is tuned to simulate winter atmospheric circulation in the extratropical hemisphere. Here we present results of HMM regime detection independently applied to four 10,000-day time series of the mid-level stream function anomalies, distributed in latitudes 36°N to 90°N with approximately 5.5×5.5 degree resolution. These non-overlapping time series are randomly taken from a very long (300,000 days) QG3-model run.

The analysis, described in Secs. 3.1-3.2 of the main article, applied to each of the four QG3 independent time series reveal 3 regimes, which are well-reproduced at HMM time steps $7 < L < 11$ days. Composite patterns of the obtained regimes shown in Fig. S2 are nearly identical for all the time series. The two most stable regimes 1 and 2 correspond to opposite, negative and positive phases of AO, respectively. Regime 1 is highly persistent, with mean lifetime of 16-18 days. Regime 2, which has mean lifetime 10-11 days, also has anomalies typical for NAO as well as high anomalies in North Pacific resembling the negative PNA pattern. The last short-lived regime 3 contains a combination of states with moderate anomalies; which can be interpreted as transient states between metastable regimes 1 and 2. It is worth noting that the characteristic timescales of QG3 model dynamics, including regime persistence, are longer than of observed behavior (see results with reanalysis data in Sec. 3). Also, its regime composition is much simpler as compared with reanalysis.

Predictability of regimes The measure of predictability of a regime A_i is expressed as:[8]

$$E(A_i,t) = \sum_j P(A_j,t|A_i,0)\log\left(\frac{P(A_j,t|A_i,0)}{\pi(A_j)}\right). \tag{S2}$$

This is the Kullback-Leibler (KL) divergence between probability distribution of regimes at time t given the regime A_i at $t = 0$, and stationary distribution π. Based on the time series of regime probabilities $p_k(t) = P(A_k|\mathbf{Y}_t)$, which are obtained from HMM learning, the conditional probabilities in Eq. S2 can be estimated as

$$P(A_j, \tau|A_i, 0) = \frac{\sum_t p_j(t+\tau)p_i(t)}{\sum_t p_i(t)}. \tag{S3}$$

Dependencies of $E(A_i,t)$ on t for each regime obtained from reanalysis are shown in Fig. S10. For comparison, in this figure we also plot the reference curves obtained under Markov assumption, where $P(A_j, \tau|A_i, 0) = (\mathbf{Q}^\tau)_{ji}$ ($\mathbf{Q}$ is the HMM transition probability matrix; see the main text).

Supplementary figures

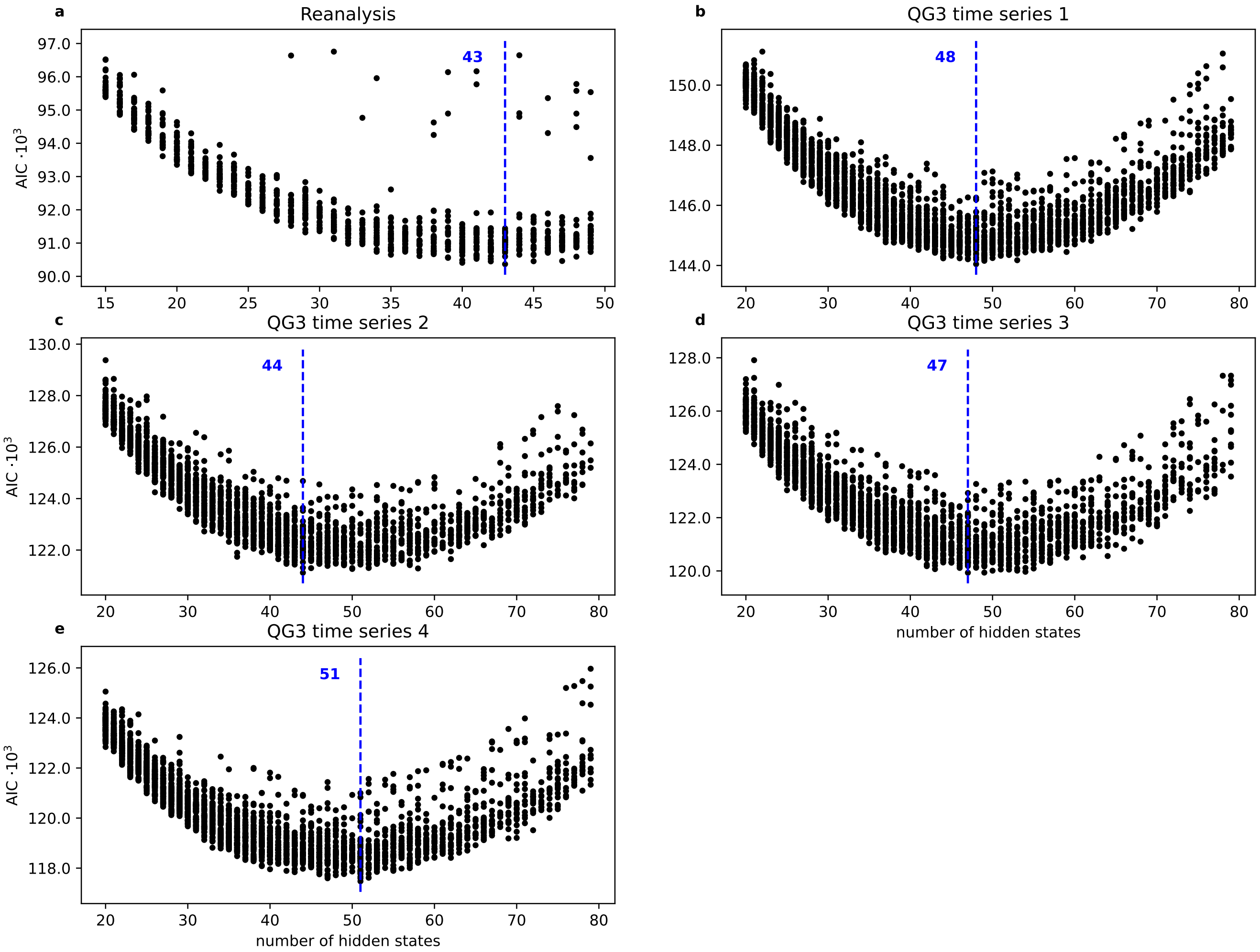

Figure S1. Optimization of the number of hidden states by Akaike Information Criterion (AIC). AIC optimality is defined as $2K - \log(P(\mathbf{Y}|\mu, K))$, where K is the number of states and $P(\mathbf{Y}|\mu, K)$ is the likelihood of HMM with parameter μ relative to the time series $\mathbf{Y}$. This value is plotted vs. K for reanalysis Z500 data (see the main article) as well as for each of the four QG3 time series. For each value of K the HMM was trained many times from different initial parameters of emission PDFs. Finally, the optimal model was selected by selecting the optimal K (blue).

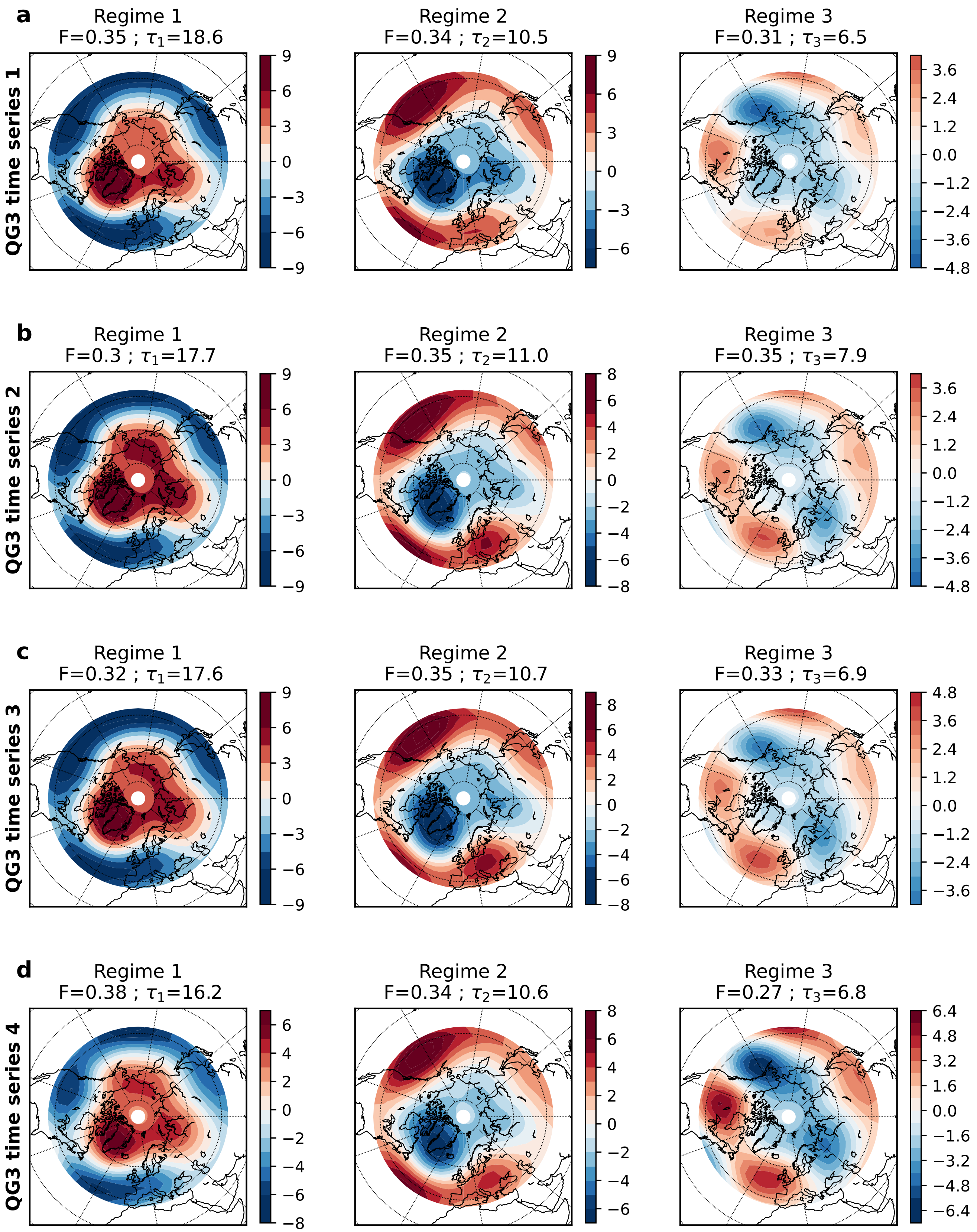

Figure S2. Regime composites for QG3 model times series. For each circulation regime the mean anomalies of the stream function from the middle level of the model are shown. The fraction of the number of days F that the system spends in each regime as well as regime mean lifetime τ are indicated.

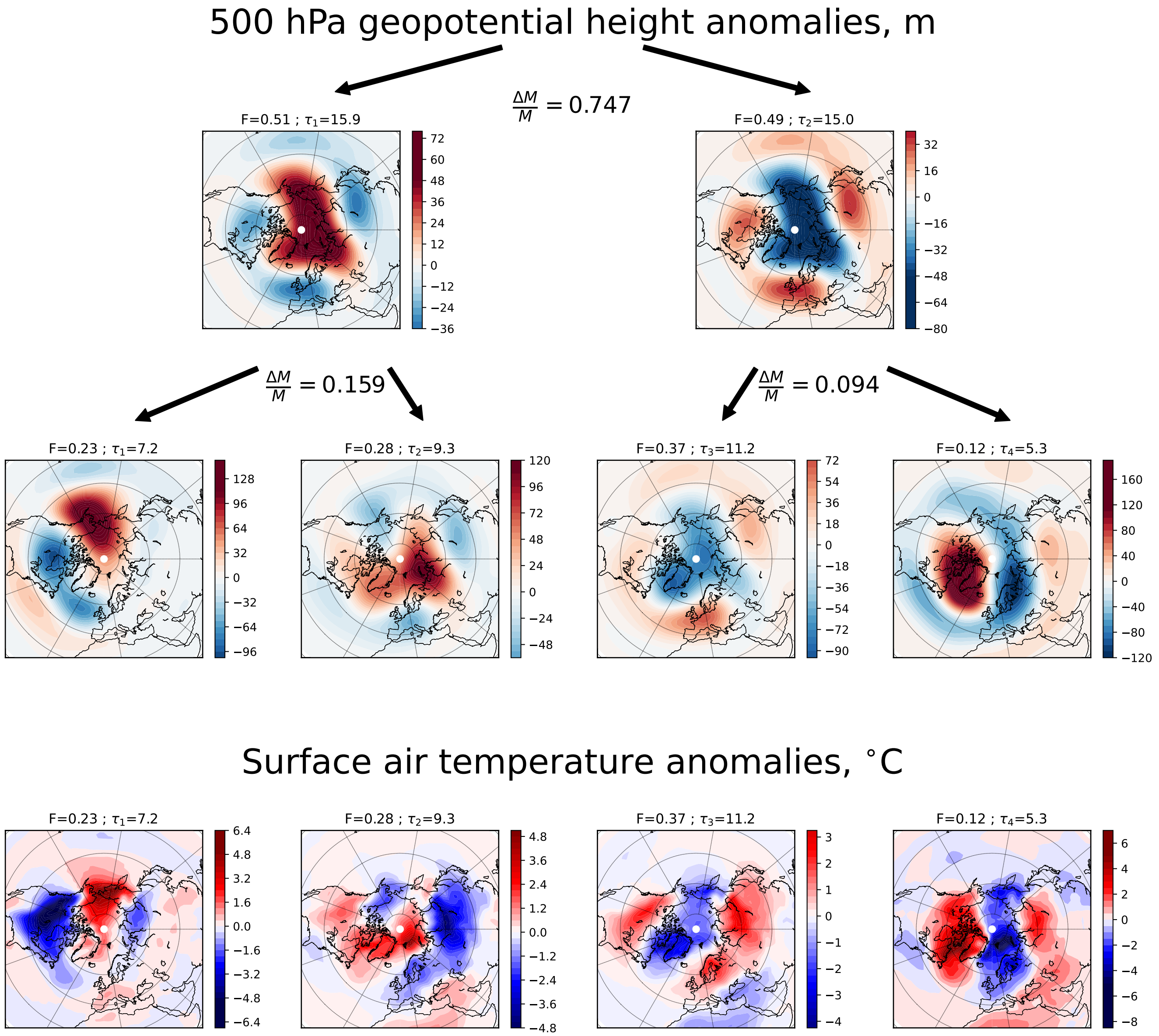

Figure S3. Composite patterns of regimes obtained with $L = 2$ days (see caption to Fig. 3 in the main article) along with the corresponding surface temperature composite.

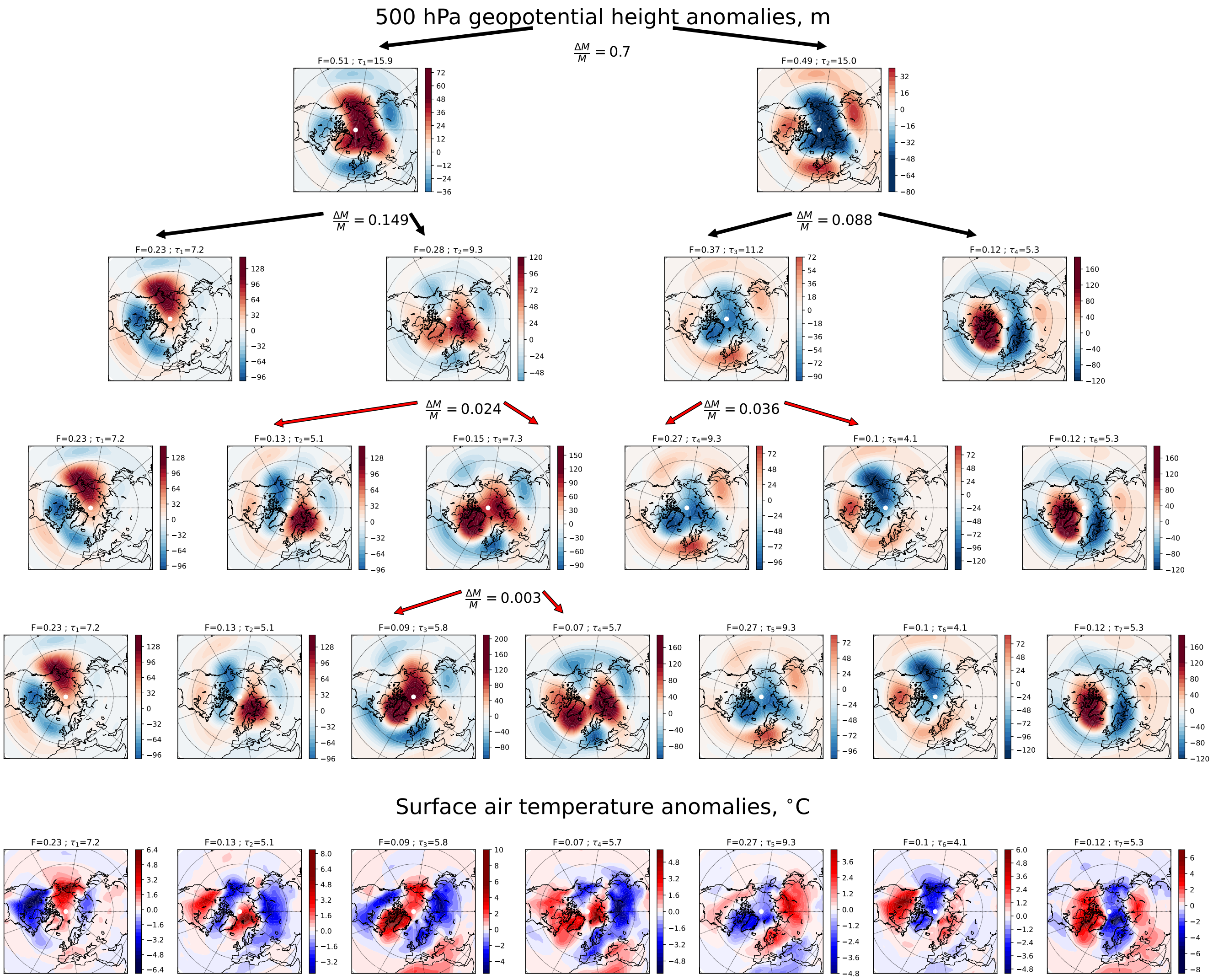

Figure S4. Same as in Fig. S3, but complemented with splits that have not passed the significance test (red arrows).

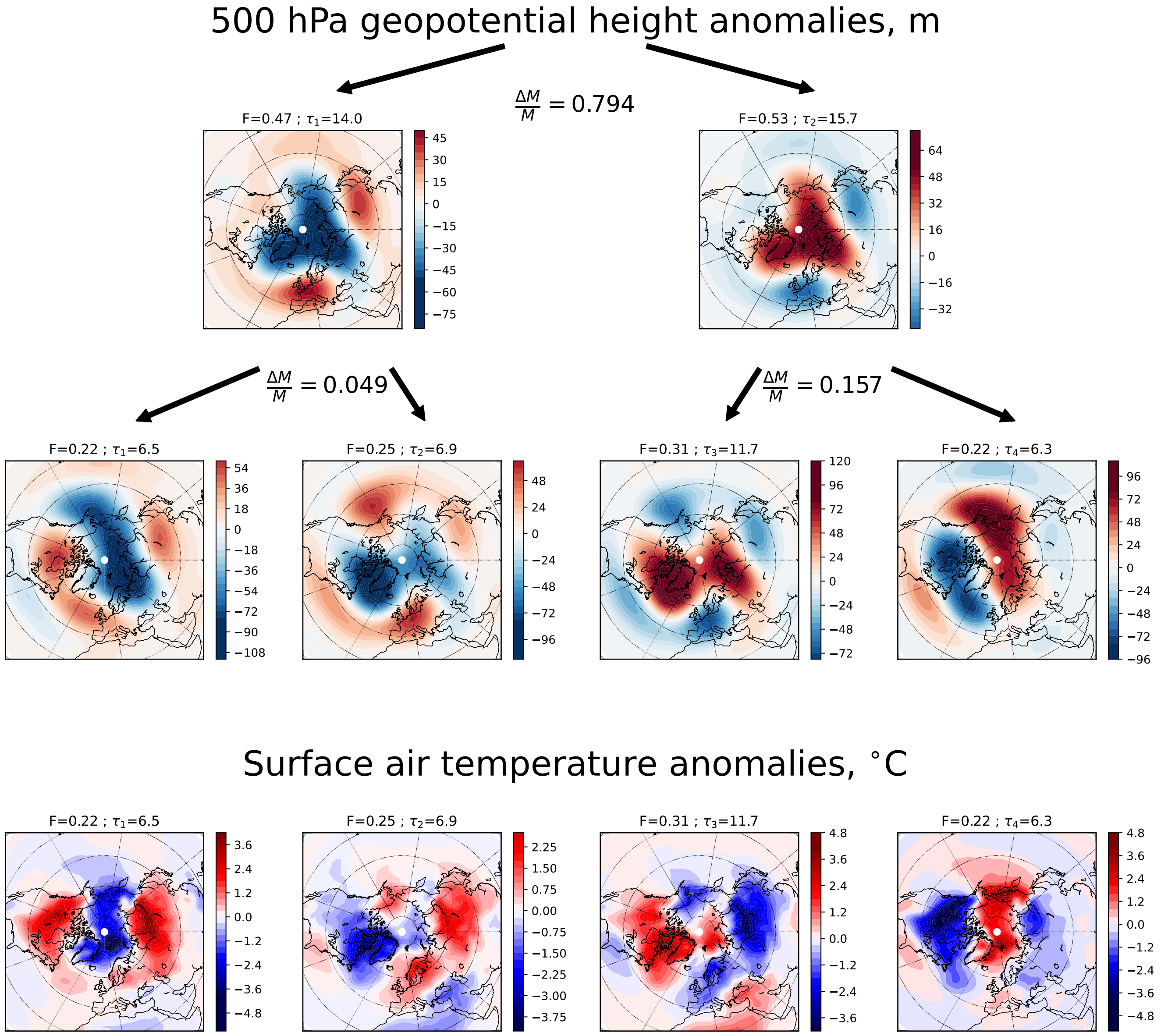

Figure S5. Same as Fig. S3, but with $L = 4$.

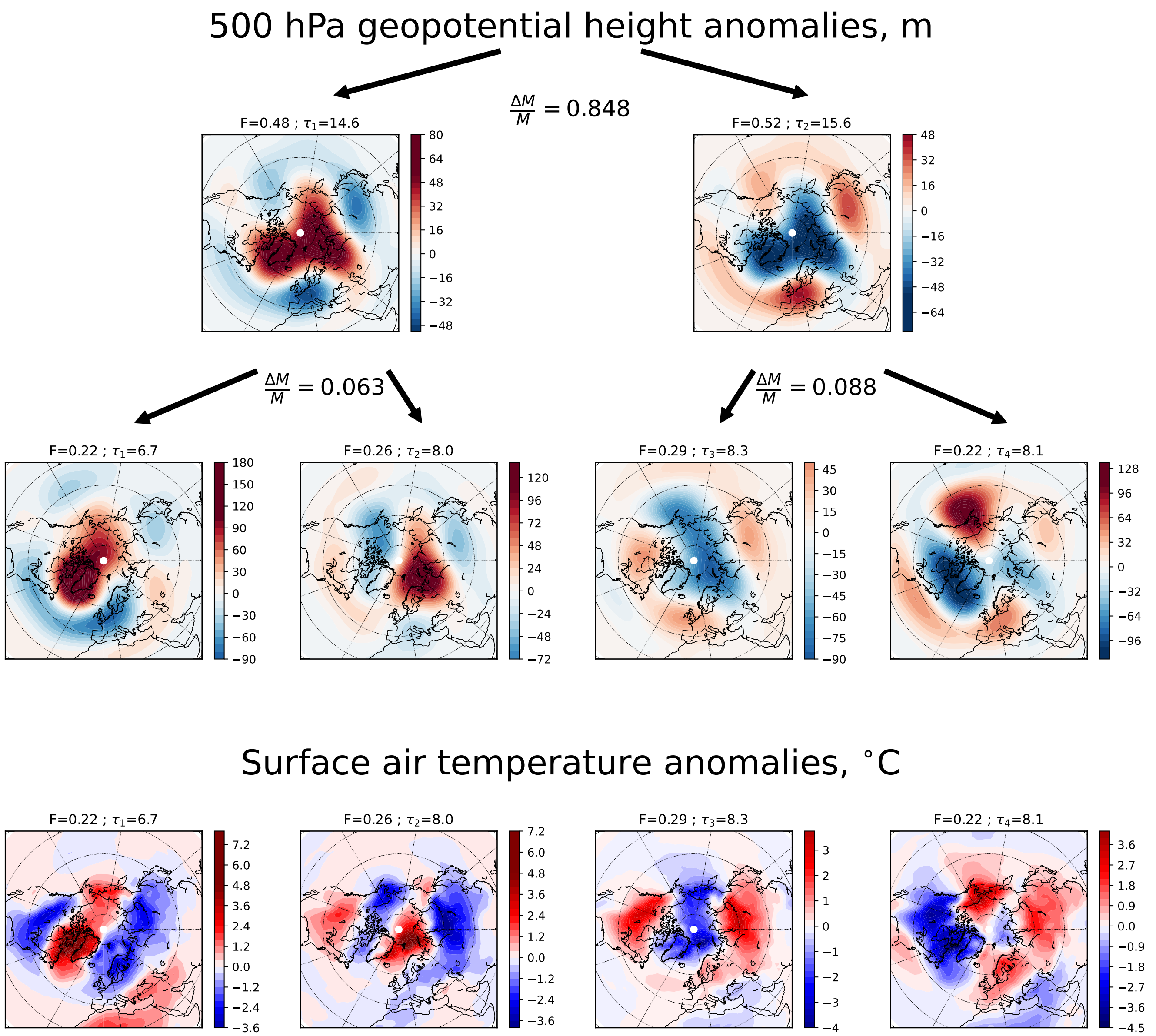

Figure S6. Same as Fig. S3, but with $L = 6$.

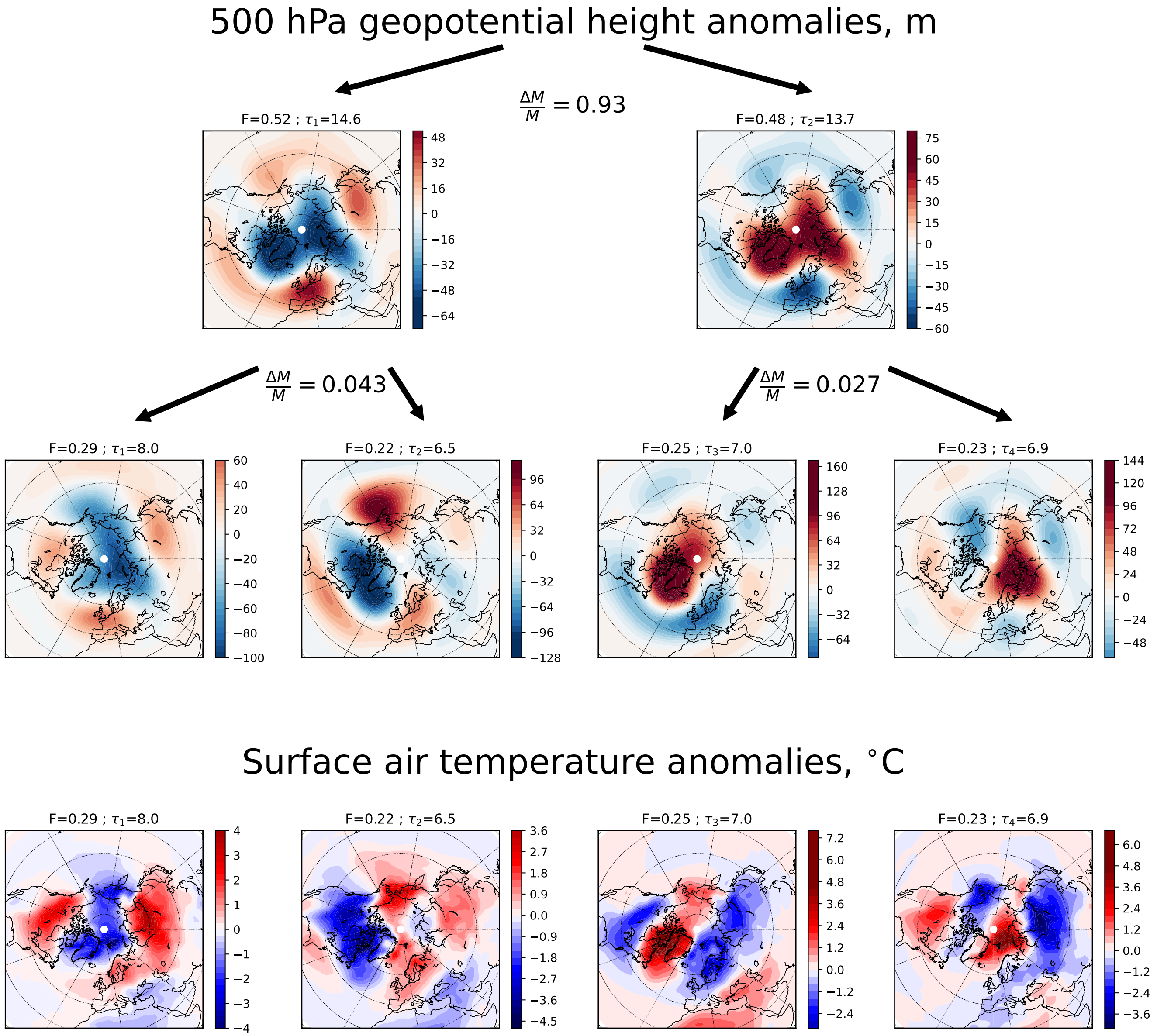

Figure S7. Same as Fig. S3, but with $L = 8$.

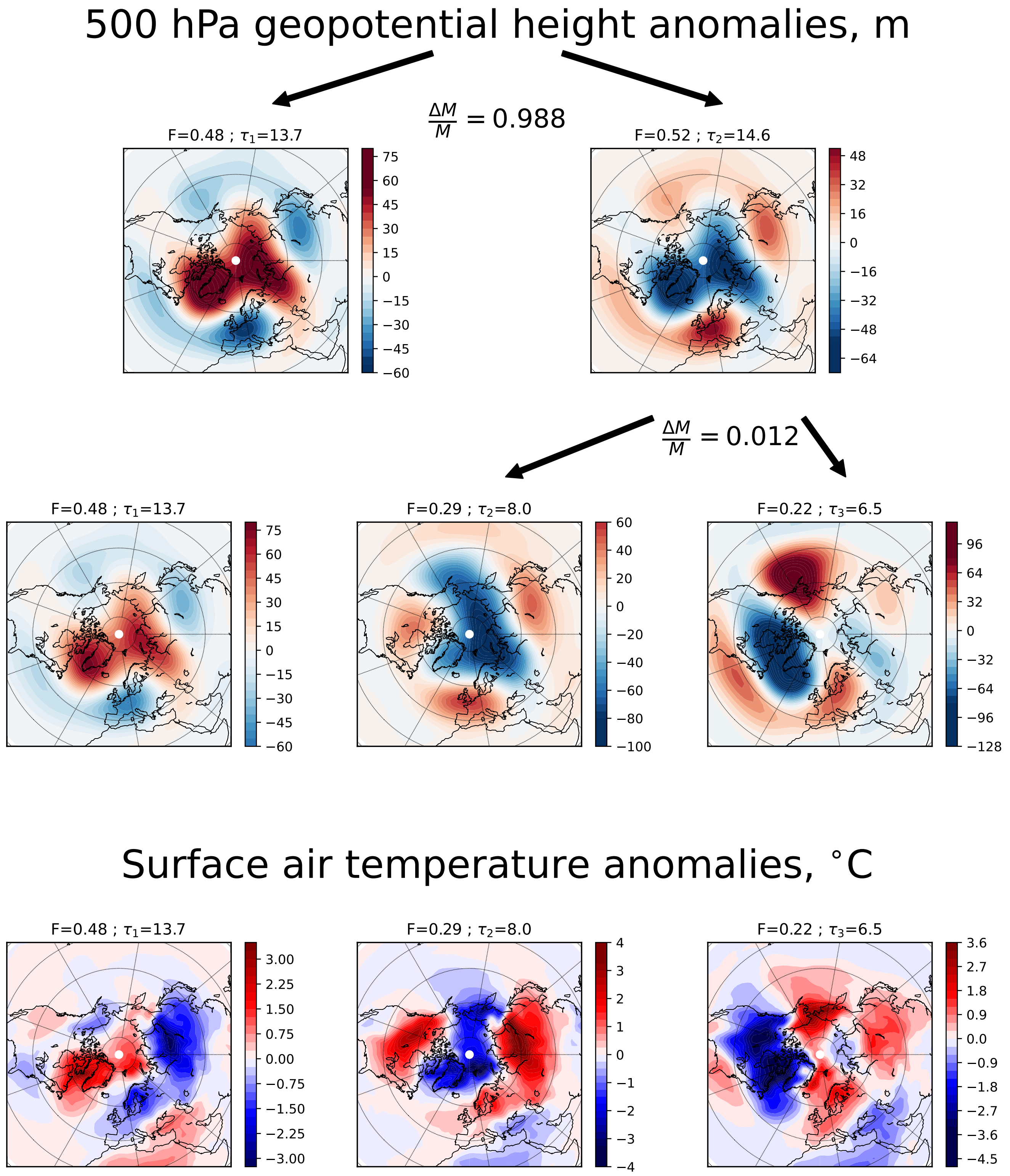

Figure S8. Same as Fig. S3, but with $L = 10$.

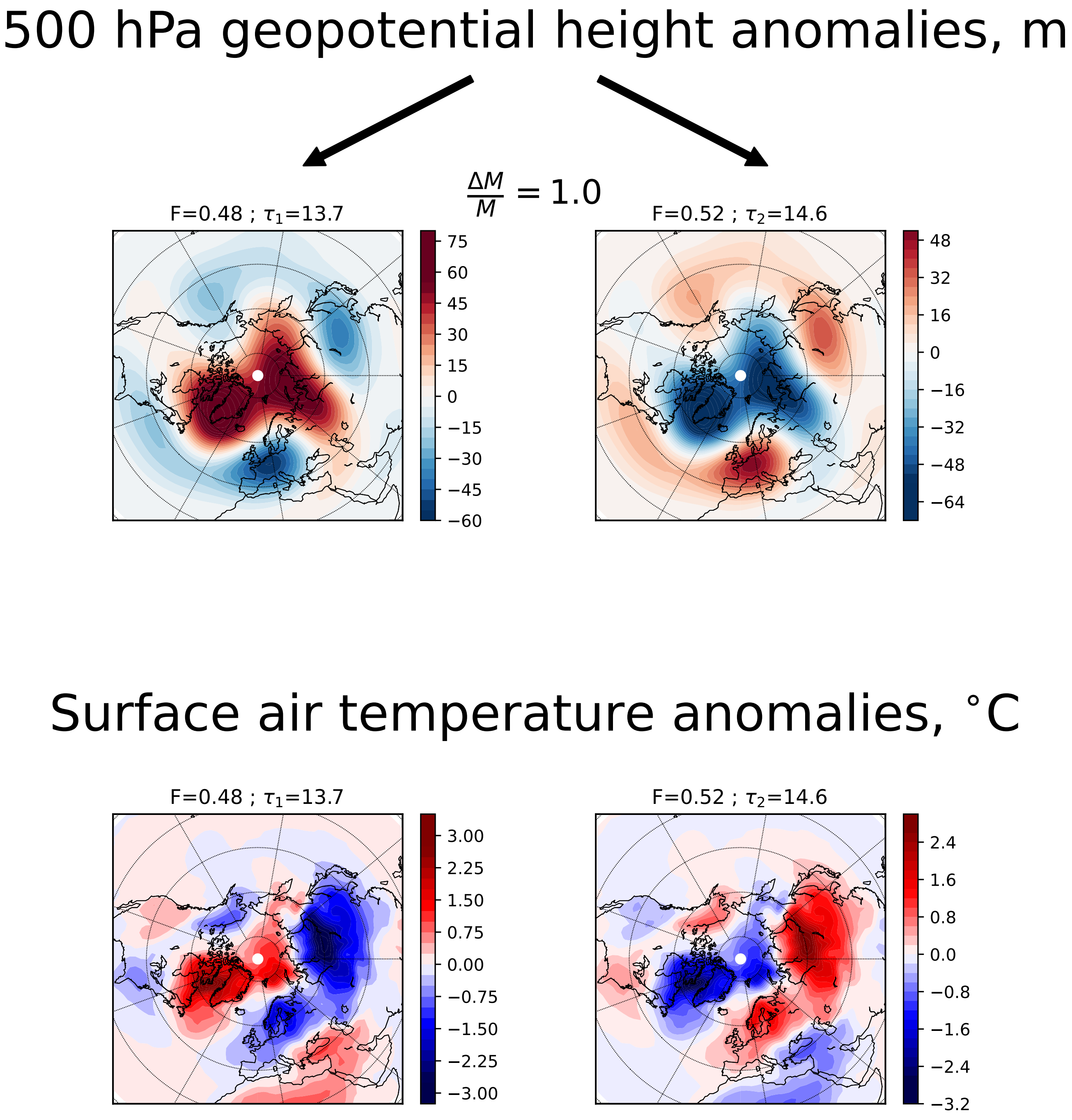

Figure S9. Same as Fig. S3, but with $L = 11$.

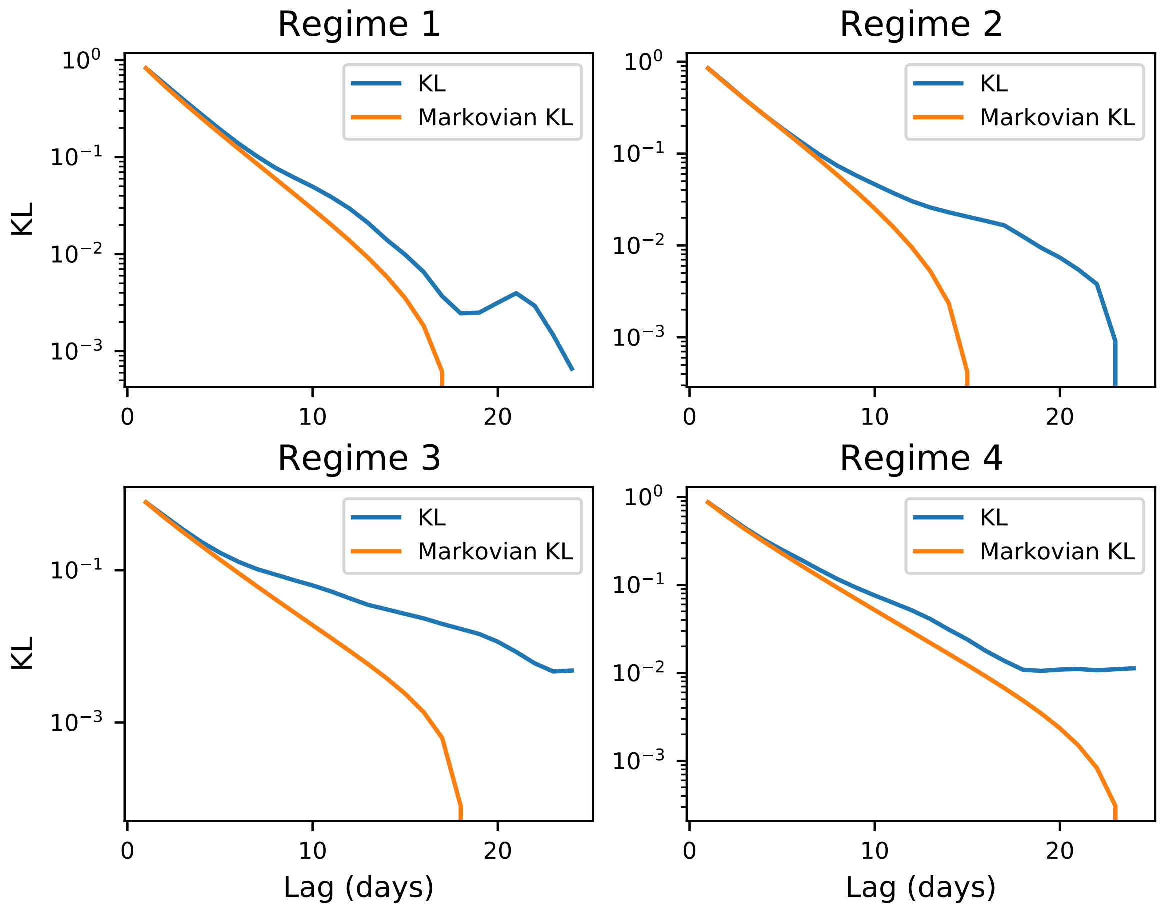

Figure S10. Measure of predictability of regimes obtained from reanalysis. See supplementary text for details.

Supplementary Files

S1 Video An example of winter atmospheric behavior including regime transitions: daily variability of Z500 anomalies during the winter 2009-2010. The animation of Z500 anomalies is shown in the main panel. The right column displays regime composites, the same as in Fig. 2 of the main manuscript. The colored frame moving across the composite figures indicates the regime that has probability greater than 95% on the current date (red, black, blue, green for regimes 1, 2, 3, 4, respectively). The current date and the corresponding regime number are displayed at the top of the figure. Regime number vs. date is plotted in the bottom panel; 0 value corresponds to lack of classification at the 95% probability threshold. Additionally, the regime number is highlighted by the colored frame.

S2 Video The same as in Animation S1, but for the winter 1991-1992.

References

1. Kondrashov, D., Ide, K. & Ghil, M. Weather regimes and preferred transition paths in a three-level quasigeostrophic model. *Journal of the Atmospheric Sciences* **61**, 568–587 (2004). URL https://doi.org/10.1175/1520-0469(2004)061<0568:WRAPTP>2.0.CO;2. https://doi.org/10.1175/1520-0469(2004)061<0568:WRAPTP>2.0.CO;2.

2. Mukhin, D., Hannachi, A., Braun, T. & Marwan, N. Revealing recurrent regimes of mid-latitude atmospheric variability using novel machine learning method. *Chaos: An Interdisciplinary Journal of Nonlinear Science* **32**, 113105 (2022). URL https://doi.org/10.1063/5.0109889. https://pubs.aip.org/aip/cha/article-pdf/doi/10.1063/5.0109889/18108937/113105_1_5.0109889.pdf.

3. Hannachi, A. & Iqbal, W. On the Nonlinearity of Winter Northern Hemisphere Atmospheric Variability. *Journal of the Atmospheric Sciences* **76**, 333–356 (2019). URL http://journals.ametsoc.org/doi/10.1175/JAS-D-18-0182.1.

4. Seleznev, A., Mukhin, D., Gavrilov, A., Loskutov, E. & Feigin, A. Bayesian framework for simulation of dynamical systems from multidimensional data using recurrent neural network. *Chaos: An Interdisciplinary Journal of Nonlinear Science* **29**, 123115 (2019). URL http://aip.scitation.org/doi/10.1063/1.5128372.

5. Marshall, J. & Molteni, F. Toward a dynamical understanding of planetary-scale flow regimes. *Journal of the Atmospheric Sciences* **50**, 1792–1818 (1993). URL https://doi.org/10.1175/1520-0469(1993)050<1792:TADUOP>2.0.CO;2. https://doi.org/10.1175/1520-0469(1993)050<1792:TADUOP>2.0.CO;2.

6. Vannitsem, S. & Nicolis, C. Lyapunov vectors and error growth patterns in a t21l3 quasigeostrophic model. *Journal of the Atmospheric Sciences* **54**, 347–361 (1997). URL https://doi.org/10.1175/1520-0469(1997)054<0347:LVAEGP>2.0.CO;2. https://doi.org/10.1175/1520-0469(1997)054<0347:LVAEGP>2.0.CO;2.

7. Corti, S., Giannini, A., Tibaldi, S. & Molteni, F. Patterns of low-frequency variability in a three-level quasi-geostrophic model. *Climate Dynamics* **13**, 883–904 (1997).

8. Vannitsem, S., Liang, X. S. & Pires, C. A. Nonlinear causal dependencies as a signature of the complexity of the climate dynamics. *Earth System Dynamics* **16**, 703–719.
